# Disruption of Spore Coat Integrity in *Bacillus subtilis* Enhances Macrophage Immune Activation

**DOI:** 10.3390/cimb47050378

**Published:** 2025-05-20

**Authors:** Bolang Liao, Yongxian Han, Zheng Wei, Xuhong Ding, Yan Lv, Xiaoqin Sun, Mingming Yang

**Affiliations:** College of Animal Science and Technology, Northwest Agriculture and Forestry University, Yangling, Xianyang 712100, China; liaobolangyx@nwafu.edu.cn (B.L.); hyx00@nwafu.edu.cn (Y.H.); weizhengwwzz@gmail.com (Z.W.); xuhongding127@163.com (X.D.); sxqxlvyan@163.com (Y.L.)

**Keywords:** *Bacillus subtilis*, spore coat, immunomodulation

## Abstract

Probiotics play a pivotal role in animal production by promoting growth, enhancing gut health, and modulating immune responses. *Bacillus subtilis*, a widely utilized probiotic, forms robust spores that exhibit exceptional resistance, making it ideal for feed applications. While *B. subtilis* spores have been shown to stimulate innate immune signaling, the specific contributions of spore coat proteins to immune modulation remain poorly characterized. In this study, we investigated the immunostimulatory effects of spores deficient in six key coat proteins: SpoIVA, SafA, CotE, CotX, CotZ, and CgeA. These proteins are essential for the assembly and structural integrity of the spore’s multi-layered coat, and are involved in recruiting other coat components. Deletion of these genes result in defects in spore coat architecture, potentially altering spore–host interactions. Using porcine alveolar macrophages (MΦ3D4/2), we assessed cytokine responses to each mutant strain. Our findings demonstrate that the absence of specific structural proteins significantly impacts immune activation, particularly through Toll-like receptor pathways. This work provides novel insights into the immunomodulatory functions of spore coat proteins and lays the foundation for the rational design of next-generation *B. subtilis*-based probiotics with enhanced immunological properties for agricultural applications.

## 1. Introduction

Probiotics are now indispensable in modern animal production systems, where they contribute to improved growth performance, gut health, and immune resilience [1,2,3]. Among them, *Bacillus subtilis* has garnered considerable attention owing to its ability to form robust, heat-resistant spores that remain viable during feed processing and storage. Emerging evidence suggests that these spores can activate innate immune pathways, notably through cytokine induction, thereby facilitating the priming of adaptive immune responses during initial antigen exposure [4]. Despite these advances, the immunological roles of specific *B. subtilis* spore coat proteins remain largely unexplored. To date, only a single report has demonstrated that CotC, a spore coat protein, can markedly upregulate IL-1β, IL-6, IL-12, and IFNγ expression in bone marrow-derived macrophages [5]. This limited insight underscores the need for a more comprehensive understanding of how spore structural components contribute to host immune modulation.

The assembly of the *B. subtilis* spore coat is a highly coordinated, gene-regulated process involving more than 80 proteins that give rise to a complex, multilayered structure. This architecture comprises a basement layer anchored to the outer forespore membrane, followed by an inner coat, outer coat, and an external crust [6,7,8]. These concentric layers are essential for spore integrity, environmental resistance, and host interactions, with a subset of proteins serving as key morphogenetic determinants. In this study, we focused on six such proteins, SpoIVA, CotE, SafA, CotX, CotZ, and CgeA, to investigate how their absence affects the immunostimulatory capacity of the spore. SpoIVA is considered the master organizer of coat assembly [9,10], with SafA and CotE directing the formation of the inner and outer coats, respectively [11,12]. CotX and CotZ contribute to the assembly of the outermost crust, whose formation depends on CotE-mediated recruitment [6,13]. CgeA, positioned within the crust, requires the coordinated action of these upstream proteins for proper localization [13]. Disruption of any of these core components compromises the architectural integrity of the spore coat, often yielding structurally defective and mechanically fragile spores [14]. Acting within a tightly coordinated assembly network, these proteins are functionally interdependent; the absence of any single factor has been shown to perturb the formation and continuity of all major coat layers including the inner, outer, and crust structures [14]. Their clearly delineated roles and essential contributions to spore morphogenesis establish a robust and experimentally accessible framework for dissecting the functional specificity of individual coat proteins.

While the structural and morphological features of *Bacillus subtilis* spore coat mutants have been well characterized, their immunological functions remain insufficiently understood. Given the central role of spore surface architecture in host–microbe interactions, elucidating how specific structural disruptions affect immune recognition is essential for advancing probiotic design. To this end, we independently constructed six targeted deletion mutants and systematically assessed their capacity to activate immune responses in porcine alveolar macrophages (Mφ3D4/2). This approach enables a controlled investigation of how alterations in spore coat composition influence innate immune signaling. Our findings aim to provide mechanistic insights into the immunomodulatory roles of spore coat components and to inform the rational development of next-generation *B. subtilis*-based bioproducts with enhanced immunostimulatory efficacy.

## 2. Materials and Methods

### 2.1. Strains and Plasmids

*B. subtilis* PY79 and *E. coli* DH5α were obtained from laboratory stock.

The plasmids used in this study included pYGJ-6, pGEM-*spoIVA*, pGEM-*cotX*, pGEM-*cgeA*, pGEM-*cotZ*, pGEM-*safA* and pGEM-*cotE*, all of which were previously constructed and archived in our laboratory. The plasmid construction scheme refers to the previous literature and has been appropriately adjusted [15]. The specific scheme is as follows: to generate the pYGJ-6 backbone, the *E. coli* plasmid pBluSKM was digested with ApaI and SacI. The spectinomycin resistance gene (*spec*) was amplified from pShuttleF using primer pair Specb-up/Specb-down. Genomic DNA from *Bacillus subtilis* PY79 served as the template for amplifying the upstream and downstream homologous arms of *clWf* and *gerDh*, using primer pairs clW-up/clW-down and gerD-up/gerD-down, respectively. Each PCR fragment (*spec*, *clWf*, and *gerDh*) was cloned into the pGEM-T Easy vector, yielding pGEM-*spec*, pGEM-*clWf* and pGEM-*gerDh*. The *spec* gene was excised from pGEM-*spec* using BamHI; *clWf* was released from pGEM-*clWf* by EcoRI and BamHI digestion; and *gerDh* was obtained from pGEM-*gerDh* via BamHI and SacI digestion. These fragments were sequentially ligated into the EcoRI–SacI-digested vector backbone to construct pYGJ-6.

In parallel, genomic DNA from *B. subtilis* PY79 was used to amplify the upstream and downstream homologous regions of *spoIVA, cotX, cgeA, cotZ, safA*, and *cotE*, using gene-specific primer pairs: *spoIVA*-fro-up/OvlapIVA-1 and OvlapIVA-2/*spoIVA*-bac-down; *cotX*-fro-up/OvlapcotX-1 and OvlapcotX-2/*cotX*-bac-down; *cgeA*-fro-up/OvlapcgeA-1 and OvlapcgeA-2/*cgeA*-bac-down; *cotZ*-fro-up/ovlapcotZ-1 and ovlapcotZ-2/*cotZ*-bac-down; *safA*-fro-up/ovlapsafA-1 and ovlapsafA-2/*safA*-bac-down; and *cotE*-fro-up/*cotE*-fro-down and *cotE*-bac-up/*cotE*-bac-down. The resulting PCR products were individually cloned into the pGEM-T Easy vector to generate the recombinant plasmids pGEM-*spoIVA*, pGEM-*cotX*, pGEM-*cgeA*, pGEM-*cotZ*, pGEM-*safA*, and pGEM-*cotE*.

### 2.2. Primers

The primers and corresponding sequences employed in this study are provided in Table 1.

### 2.3. Construction of Recombinant Vectors and Mutant Strains

A gene deletion mutant of *Bacillus subtilis* PY79 was constructed using a homologous recombination strategy, following the classic homologous recombination method [16]. The detailed information of recombinant plasmids targeting spore coat protein genes (*spoIVA*, *cotX*, *cgeA*, *cotZ*, *safA*, *cotE*) is shown in Tables S1 and S2. The following is a detailed method:

(1) Genomic DNA extraction and amplification of homology arms

Genomic DNA from *B. subtilis* PY79 was extracted and used as a template for PCR amplification of the upstream and downstream homologous arms of each target gene. Primers were designed to incorporate BamH I restriction sites for downstream cloning.

(2) Fusion of homology arms by overlap extension PCR

Overlap extension PCR was performed using high-fidelity polymerases to assemble the flanking regions of each gene into continuous fragments.

(3) Initial cloning and screening

The fused PCR products were ligated into the pGEM-T Easy vector. Recombinant plasmids were selected by blue/white screening and validated by restriction digestion and PCR. All reactions were carried out at room temperature for 1 h.

(4) Insertion of antibiotic resistance marker

The spectinomycin resistance gene (*spec*) was amplified from plasmid pYGJ-6 and inserted into linearized constructs (pGEM-*spoIVA*, pGEM-*cotX*, pGEM-*cgeA*, pGEM-*cotZ*, pGEM-*safA*, pGEM-*cotE*). The ligated vectors pB-*spoIVA*, pB-*cotX*, pB-*cgeA*, pB-*safA*, pB-*cotZ* and pB-*cotE* were obtained. The ligated vectors were transformed into *E. coli* DH5α, and transformants were selected on LB agar plates containing 50 μg/mL spectinomycin. Positive clones were verified by PCR and sequencing.

(5) Transformation into *B. subtilis*

Verified integration vectors were linearized with either Pst I or Sal I, depending on the construct design, and transformed into competent *B. subtilis* PY79 cells. Spectinomycin-resistant colonies were selected on LB agar plates supplemented with 50 μg/mL spectinomycin.

(6) Verification of mutants

Genomic DNA from spectinomycin-resistant *B. subtilis* colonies was extracted and subjected to PCR using gene-specific primers. Amplified fragments were analyzed by agarose gel electrophoresis and Sanger sequencing to confirm successful gene deletions.

### 2.4. Growth Curve Analysis

All *B. subtilis* strains were initially streaked on LB agar plates and incubated at 37 °C for 12 h. Single colonies displaying uniform morphology were inoculated into 30 mL of LB broth in Erlenmeyer flasks and cultured at 37 °C with agitation at 220 rpm. Bacterial growth was monitored by measuring the optical density at 600 nm (OD_600_) at 2-h intervals throughout the growth phase.

### 2.5. Assessment of Sporulation Efficiency

To evaluate sporulation capacity, strains were streaked onto LB agar plates and incubated at 37 °C for 12 h. Colonies of similar size (approximately 1 mm) were selected and inoculated into 5 mL LB broth, followed by incubation at 37 °C with shaking at 220 rpm for 12 h. Subsequently, a 1:100 dilution was transferred into 30 mL of Difco Sporulation Medium (DSM) and cultured at 37 °C, undergoing shaking at 200 rpm until 16 h after the onset of stationary phase to induce sporulation [17]. Samples were collected at defined time points and subjected to Gram staining to visualize spore formation. Sporulation efficiency was evaluated via phase-contrast microscopy (Olympus, Tokyo, Japan) based on the presence and abundance of mature spores.

### 2.6. Spore Preparation

Sporulation was induced as described above. Following incubation, cultures were harvested by centrifugation at 8000 rpm for 20 min, and the resulting pellets were washed twice with sterile phosphate-buffered saline (PBS). The spore pellets were resuspended in 30 mL PBS. To account for the potentially reduced lysozyme resistance of coat-deficient mutants, spores from wild-type and mutant strains were purified using linear 25–55% Urografin (Bayer, Leverkusen, Germany) density-gradient centrifugation, rather than enzymatic treatment [18]. The purified spores were sequentially washed with 1 M sterile NaCl and 1 M sterile KCl to remove residual debris, followed by final resuspension in sterile PBS. Spore suspensions were standardized to 1 × 10^7^ CFU/mL and stored at −20 °C until use.

### 2.7. Transmission Electron Microscopy (TEM)

Transmission electron microscopy (TEM) observations were performed based on previously reported protocols [19], with minor modifications to optimize sample staining and visualization. For ultrastructural analysis, spore suspensions were centrifuged and the pellets fixed overnight at 4 °C in 2.5% glutaraldehyde prepared in PBS. Fixed samples were washed three times in PBS (15 min per wash) and subsequently post-fixed in 1% osmium tetroxide for 2 h. Following additional PBS washes, samples were dehydrated through a graded ethanol series (30%, 50%, 70%, 80%, 90%, and 100%), each step lasting 30 min, and subjected to three final washes in 100% ethanol. Dehydrated samples were embedded in epoxy resin, pre-polymerized at 45 °C for 24 h, and fully polymerized at 60 °C for another 24 h. Ultrathin sections were prepared, stained, and imaged using a Hitachi H7650 transmission electron microscope (Hitachi High-Technologies Corporation, Tokyo, Japan) operated at 80 kV.

### 2.8. Cell Culture

The porcine alveolar macrophage cell line Mφ3D4/2 was obtained from laboratory stocks. Cells were rapidly thawed in a 37 °C water bath and immediately resuspended in RPMI-1640 medium (Thermo Fisher Scientific, Waltham, MA, USA). Following centrifugation, the cell pellet was resuspended in fresh RPMI-1640 medium and cultured at 37 °C in a humidified incubator with 5% CO_2_. Upon reaching ~80% confluency, cells were harvested by trypsinization, centrifuged, and resuspended to a final concentration of 1 × 10^4^ cells/mL. For co-culture assays, cells were seeded in 6-well plates (2 mL per well) and incubated with spores of wild-type or mutant *B. subtilis* strains (1 × 10^7^ CFU/mL) for 12 h [20]. Experimental controls included untreated cells (blank), cells treated with wild-type spores, and a positive control group stimulated with lipopolysaccharide (LPS, 100 ng/mL). Each condition was tested in triplicate and repeated independently three times.

### 2.9. RNA Extraction and RT–qPCR

Total RNA was isolated from Mφ3D4/2 cells using an RNA extraction kit (Thermo Fisher Scientific, Waltham, MA, USA), and RNA concentrations were quantified using a NanoDrop spectrophotometer (Thermo Fisher Scientific, Waltham, MA, USA). Residual genomic and plasmid DNA was eliminated with Turbo DNase I treatment (Thermo Fisher Scientific, Rockford, IL, USA). Reverse transcription was carried out using 1 μg of total RNA in a 20 μL reaction, as described previously [21].

Quantitative real-time PCR (qRT–PCR) was performed in 10 μL reaction volumes comprising 5 μL iQ SYBR Green Supermix (Bio-Rad, Hercules, CA, USA), 3 μL nuclease-free water, 0.5 μL each of forward and reverse primers (10 μM; see Table S3), and 1 μL of cDNA template. Gene expression analysis [22] targeted TLR2, TLR4, TLR9, MyD88, IRAK1, and NF-κB2, employing the ΔΔCt method for relative quantification [23]. Expression levels were normalized to the housekeeping gene GAPDH, used as the internal control. All reactions were performed in technical triplicates across three biological replicates. Data are presented as mean ± SD (standard deviation), and statistical analysis was conducted using one-way ANOVA followed by Bonferroni’s test.

### 2.10. ELISA

Cytokine levels in cell culture supernatants were quantified using an ELISA kit (Thermo Fisher Scientific, Waltham, MA, USA) following the manufacturer’s instructions. Standards, samples, and diluents were prepared accordingly. The samples and standards were added to ELISA plates pre-coated with specific antibodies and incubated at 37 °C for 1 h. Following incubation, enzyme-conjugated secondary antibodies were added and incubated at 37 °C for an additional 1 h, after which the plates were washed four times. Substrate solution was subsequently added, and the reaction was terminated upon the development of color. Absorbance was measured at the specified wavelength using a microplate reader (Bio-Rad, Hercules, CA, USA), and cytokine concentrations were determined by comparison to the standard curve.

### 2.11. Statistical Analyses

Statistical analyses were conducted using GraphPad Prism (v9.5.1, GraphPad Software Inc., San Diego, CA, USA). Differences between groups were assessed using one-way or two-way analysis of variance (ANOVA), as appropriate. A value of *p* < 0.05 was considered statistically significant. All experiments were independently performed at least three times.

## 3. Results

### 3.1. Construction and Validation of Spore Coat Gene Deletion Mutants

**Figure 1:**
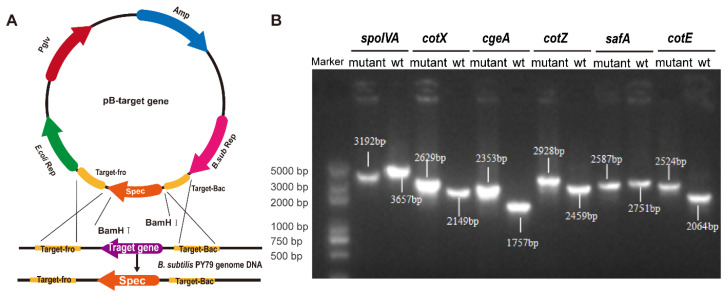
Construction and identification of spore coat protein deletion mutants. (**A**) Schematic diagram illustrating the homologous recombination vector used to generate the mutant strains. (**B**) PCR identification of knockout strains using primers targeting the homologous arms. The successful gene deletions are confirmed by the presence of product bands of different sizes.

Targeted gene deletions were achieved via homologous recombination using plasmid-based constructs designed to disrupt key spore coat protein genes (Figure 1A). Six mutant strains of *B. subtilis*, *spoIVA^−^* mutant (Δ*spoIVA*), *cotX^−^* mutant (Δ*cotX*), *cgeA^−^* mutant (Δ*cgeA*), *cotZ^−^* mutant (Δ*cotZ*), *safA^−^* mutant (Δ*safA*), and *cotE^−^* mutant (Δ*cotE*), were successfully generated. PCR analysis confirmed the integration of the spectinomycin resistance cassette at the intended genomic loci, with amplicon sizes consistent with predicted outcomes (Figure 1B). Sanger sequencing and alignment against NCBI reference genomes verified precise gene disruption, with all constructs displaying > 99% sequence identity, thereby confirming the fidelity of the recombination events.

### 3.2. Phenotypic Characterization of Spore Coat Protein Mutants

To ensure the suitability of the constructed mutants for downstream immunological assays, we performed a comprehensive phenotypic assessment, including growth dynamics, sporulation efficiency, and ultrastructural analysis of spores.

(1) Growth dynamics

**Figure 2:**
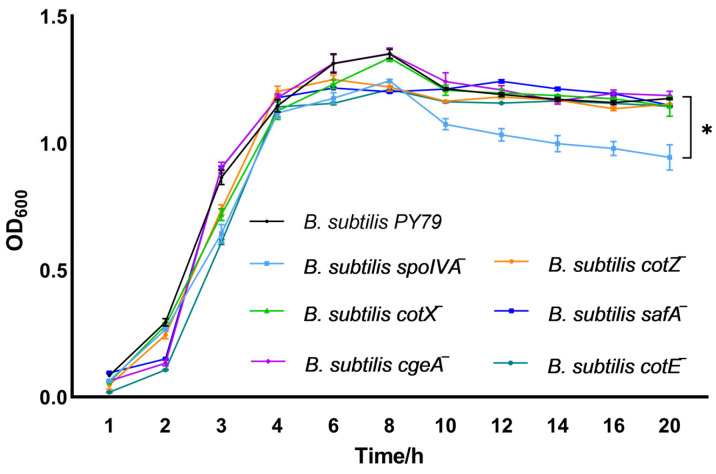
Growth dynamics of spore coat protein-deficient *Bacillus subtilis* mutants. Growth curves of spore coat protein-deficient mutant strains were monitored by measuring optical density at 600 nm (OD_600_) at hourly intervals starting from 1 h post-inoculation. Growth kinetics were initiated by inoculating LB broth with single colonies to a starting OD600 of 0.05 ± 0.02. Bacterial cultures were incubated at 37 °C with shaking at 200 rpm in LB medium. Data represent the mean ± standard deviation (SD) of three independent biological replicates. Statistical analysis was performed using two-way ANOVA followed by Bonferroni’s multiple comparison test (* *p* < 0.05).

Growth kinetics analysis revealed that gene deletion of *spoIVA* markedly impaired the proliferation of *Bacillus subtilis*, highlighting its essential role in sustaining vegetative growth (Figure 2). By contrast, gene deletion of *cotX*, *cgeA*, *cotZ*, *safA*, or *cotE* had minimal impact, as the corresponding mutants exhibited growth dynamics similar to the wild-type strain.

(2) Sporulation efficiency

Sporulation efficiency was evaluated using Gram staining and microscopic observation (Figure S1). The *spoIVA^−^* mutant displayed markedly reduced spore formation, consistent with its critical function in early morphogenetic events [24]. All other mutants formed spores at levels similar to the wild type, suggesting that disruption of these specific spore coat proteins does not grossly impair sporulation or spore recovery.

(3) Ultrastructural analysis by transmission electron microscopy (TEM)

**Figure 3:**
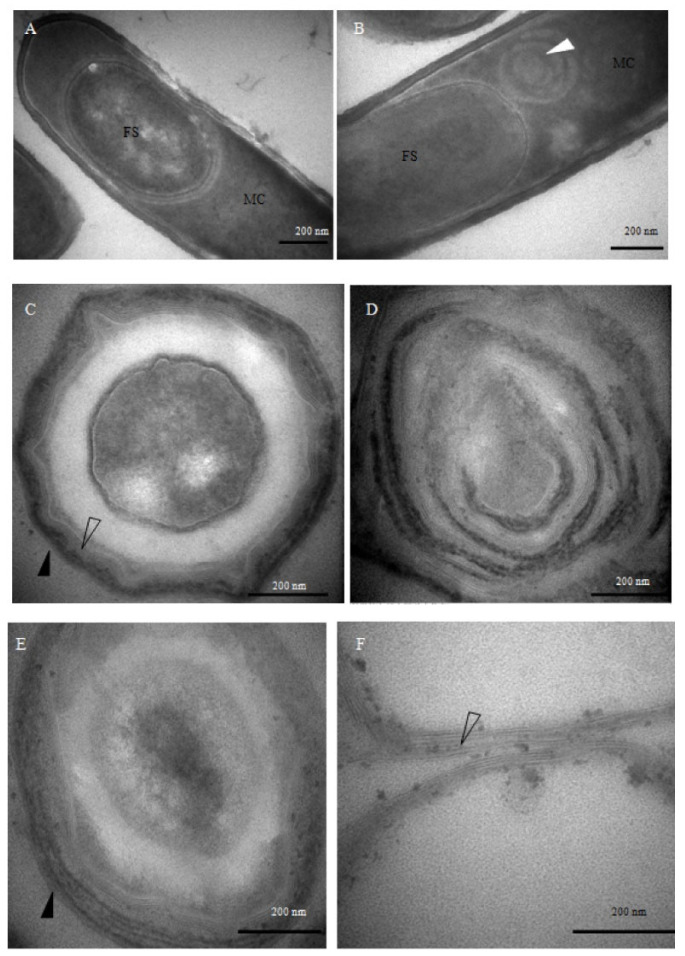
Transmission electron microscopy (TEM) analysis of mutant sporulation and spore coat morphology. (**A**) Wild-type *Bacillus subtilis* cells after 10 h of sporulation. (**B**) *spoIVA^−^* mutant cells after 10 h of sporulation. (**C**) Wild-type spores after 48 h of sporulation. (**D**) *spoIVA^−^* mutant spores after 48 h of sporulation. (**E**) *safA^−^* mutant spores after 48 h of sporulation. (**F**) *cotE^−^* mutant spores after 48 h of sporulation. FS: forespore; MC: mother cell. White arrows indicate the whirl-like structures formed by spore coat proteins, hollow arrows indicate the inner coat layer, and black arrows highlight the outer coat layer. Bars in panels (**A**–**F**), 500 nm.

TEM imaging revealed pronounced structural alterations in the spore coats of the mutant strains (Figure 3). Ten hours after the onset of sporulation, morphologically intact sporophytes were observed in the wild-type strain (Figure 3A). In contrast, the *spoIVA^−^* mutant exhibited aberrant ‘whirlpool-like’ structures encircling the forespore (Figure 3B), and by 48 h, its architecture had become markedly disorganized (Figure 3D). These phenotypes are consistent with previously reported defects in spore coat localization and vortex formation associated with *spoIVA^−^* mutants [25], in contrast to the well-structured spores formed by the wild-type *B. subtilis* (Figure 3C). In the *safA^−^* mutant, the inner coat appeared attenuated and less defined, while the outer coat was visibly disordered (Figure 3E). Spores derived from the *cotE*⁻ mutant retained a well-defined inner coat but exhibited a marked absence of the outer coat layer (Figure 3F), underscoring the indispensable role of CotE in outer coat morphogenesis, rather than in the assembly of other coat structures. Together, these observations demonstrate that the deletion of distinct coat protein genes leads to discrete alterations in spore architecture.

### 3.3. Mutant Spores Differentially Activate TLR and Inflammatory Responses

**Figure 4:**
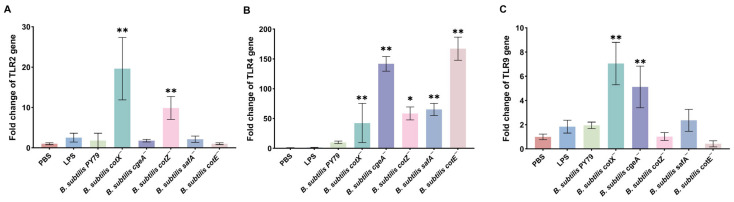
Gene expression of Toll-like receptors (TLRs) in macrophages stimulated with various *Bacillus subtilis* mutant strains. (**A**) Relative expression of TLR2 in porcine alveolar macrophages (MΦ3D4/2) following stimulation with different mutant strains. (**B**) Relative expression of TLR4 in porcine alveolar macrophages (MΦ3D4/2) following stimulation with different mutant strains. (**C**) Relative expression of TLR9 in porcine alveolar macrophages (MΦ3D4/2) following stimulation with different mutant strains. mRNA levels were quantified by qRT-PCR and normalized to the housekeeping gene GAPDH. Data represent the mean ± standard deviation (SD) of three independent biological replicates. Statistical analysis was performed using one-way ANOVA followed by Bonferroni’s multiple comparison test (* *p* < 0.05, ** *p* < 0.01). Statistical significance was determined by comparison of PBS treatment with all other treatments.

To elucidate the impact of spore coat mutations on innate immune signaling, we quantified the mRNA expression of key pattern recognition receptors and inflammatory mediators in porcine alveolar macrophages (Mφ3D4/2) using qRT-PCR. All five mutants induced a significant upregulation of TLR4 expression compared to untreated controls, with expression levels markedly surpassing those of TLR2 and TLR9 (Figure 4B), suggesting that TLR4 signaling serves as the principal axis of macrophage activation by mutant spores. Notably, the *cotX*^−^ and *cotZ*^−^ mutants also elicited a moderate but significant increase in TLR2 transcript levels (Figure 4A), while *cotX*^−^ and *cgeA*^−^ mutants triggered elevated TLR9 expression (Figure 4C), indicating partial activation of these alternative TLR pathways. These findings imply that the disruption of the outermost crust layer, comprising CotX, CotZ, and CgeA, relieves a repressive effect on TLR-mediated signaling, thereby enhancing receptor responsiveness.

**Figure 5:**
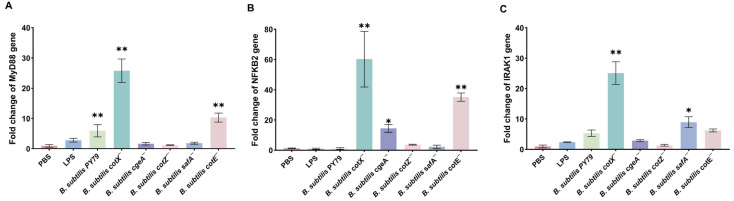
Expression of key immune signaling molecules in macrophages stimulated with *Bacillus subtilis* mutant strains. (**A**) Relative mRNA expression of MyD88 in porcine alveolar macrophages (MΦ3D4/2) following stimulation with different mutant strains. (**B**) Relative mRNA expression of NF-κB2 in porcine alveolar macrophages (MΦ3D4/2) following stimulation with different mutant strains. (**C**) Relative mRNA expression of IRAK1 in porcine alveolar macrophages (MΦ3D4/2) following stimulation with different mutant strains. Total RNA was extracted after 6 h of co-culture, and transcript levels were quantified by qRT-PCR using GAPDH as the internal control. Data are presented as mean ± standard deviation (SD) from three independent biological replicates. Statistical analysis was performed using one-way ANOVA followed by Bonferroni’s multiple comparison test. (* *p* < 0.05, ** *p* < 0.01). Statistical significance was determined by comparison of PBS treatment with all other treatments.

Consistent with heightened TLR activation, qRT-PCR analysis revealed that spores from cotX^−^ and cotE^−^ mutants significantly elevated transcription of the key adaptor protein MyD88 and effector NF-κB2 (*p* < 0.05) (Figure 5A,B). Moreover, cotX^−^ and safA^−^ mutants induced a marked increase in IRAK1 expression (Figure 5C). These results suggest that coat and crust layer proteins, particularly CotX, may act as immunomodulatory elements that attenuate pro-inflammatory signaling. Disruption of these structural components lifts this suppression, resulting in heightened macrophage activation via MyD88-dependent pathways.

### 3.4. Effect of Spore Coat Protein Mutants on the Expression of Inflammatory Cytokines

**Figure 6:**
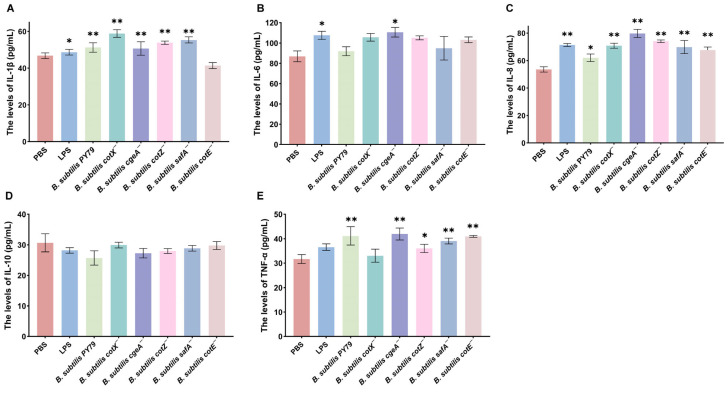
Inflammatory cytokine responses in macrophages stimulated with *Bacillus subtilis* mutant strains. Porcine alveolar macrophages (MΦ3D4/2) were co-cultured with individual mutant strains for 6 h, and culture supernatants were subsequently collected for cytokine quantification via ELISA. (**A**) IL-1β levels. (**B**) IL-6 levels. (**C**) IL-8 levels. (**D**) TNF-α levels. (**E**) IL-10 levels. Data represent the mean ± standard deviation (SD) of three independent biological replicates. Statistical significance was determined using one-way ANOVA followed by Bonferroni’s multiple comparison test (* *p* < 0.05, ** *p* < 0.01). Statistical significance was determined by comparison of PBS treatment with all other treatments.

To investigate the impact of spore coat protein mutations on inflammatory cytokine production, macrophages were stimulated with the mutant strains, and cytokine levels were quantified. Transcriptomic analysis revealed that treatment with *Bacillus subtilis cotX*^−^, *cgeA*^−^, *cotZ*^−^, and *safA*^−^ mutants significantly upregulated IL-1β expression in porcine alveolar macrophages (3D4/2), with increases of 30.71%, 20.47%, 17.32%, and 26.77%, respectively, relative to the PBS control (*p* < 0.05) (Figure 6A). Notably, the *cgeA^−^* mutant also markedly enhanced IL-6 expression (17.12% increase; *p* < 0.05), suggesting a broader pro-inflammatory potential (Figure 6B). Similarly, IL-8 transcription was significantly elevated following treatment with *cotX*^−^, *cgeA*^−^, *cotZ*^−^, *safA*^−^, and *cotE^−^* mutants, with respective increases of 25.90%, 39.76%, 32.5%, 25.9%, and 20.48% (*p* < 0.05; Figure 6C). In addition, TNF-α expression was significantly upregulated by *cgeA*^−^, *cotZ*^−^, *safA*^−^, and *cotE*^−^ mutants, with respective increases of 32.61%, 15.22%, 21.74%, and 31.52% (*p* < 0.05; Figure 6D). In contrast, no significant changes in IL-10 levels were observed in macrophages treated with the LPS, wild-type, or mutant strains (Figure 6E). These results indicate that specific spore coat proteins, including CotX, CotZ, CgeA, and SafA, play a key role in modulating pro-inflammatory cytokine expression, potentially suppressing the activation of certain inflammatory responses.

## 4. Discussion

So far, the probiotic effects of *Bacillus subtilis* remain incompletely understood, with emerging evidence suggesting that these effects may be mediated by metabolites produced by the microorganism itself [26]. The spore germination process, a crucial step for the activation of *B. subtilis* cells, has been closely linked to cellular activity [27]. The spore coat of *B. subtilis* is essential for both spore formation and function, comprising a highly intricate multi-layered structure that includes the basal layer, cortex, inner coat, outer coat, and the external crust [6]. The assembly of this spore coat involves over 80 proteins that self-organize into distinct layers. SpoIVA, CotE, SafA, CotX, CotY, CotZ, and CgeA act as key proteins involved in this process [28]. SpoIVA, CotE, and SafA are key morphogenetic proteins essential for spore coat formation and proper localization of coat layers [29]. CotX, CotZ, and CgeA represent outer coat or crust-associated proteins that are known to influence spore surface properties, including interaction with host cells [30,31]. Their functional diversity and surface exposure make them ideal candidates for investigating how specific coat components modulate host immune responses. In this study, we systematically disrupted key genes encoding spore coat proteins and assessed the resulting mutations’ effects on immune responses induced in macrophages.

SpoIVA plays a pivotal role in orchestrating the assembly of the spore cortex and coat, functioning at the critical interface between these two layers [32]. Loss of SpoIVA results in the formation of spores with incomplete and disorganized coats, severely compromising spore morphogenesis. CotE, a key determinant of inner coat assembly, facilitates the recruitment of outer coat proteins through its C-terminal domain [33,34]. Spores lacking CotE exhibit an absence of outer coat components and a reduced inner coat thickness, yet retain intact growth and sporulation capacity [12]. SafA acts as a structural scaffold bridging the cortex and outer coat, with its N-terminal region anchored at the cortex–coat interface and a C-terminal domain homologous to other inner coat proteins [35,36]. Deletion of SafA leads to inner coat thinning and disorganization, although spore development, including growth, sporulation, and germination, remains largely unaffected. The outermost crust layer is predominantly composed of CotX, CotY, and CotZ, which are indispensable for coat glycosylation and maintenance of surface architecture, with CgeA localization dependent on these structural proteins [37,38,39]. Mutants deficient in these components display thinner coats and altered surface morphology, but exhibit no significant defects in vegetative growth or sporulation efficiency [9,12,40]. These phenotypes are consistent with our current observations, confirming the successful generation of structurally distinct mutant strains.

Emerging evidence suggests that spore germination of *Bacillus anthracis* may occur extracellularly within pulmonary tissues [41]. Alveolar macrophages are central to the host’s initial defense against anthrax, orchestrating microbicidal responses against intracellular pathogens and contributing to early clearance of the infection [42]. Intriguingly, *B. subtilis* spores have been shown to potentiate innate immune responses, offering protection against respiratory pathogens [43,44]. These spores can stimulate the production of pro-inflammatory cytokines, including IL-1β and IL-6, in innate immune cells such as monocytes and macrophages [45]. Despite these findings, the immunomodulatory effects of *B. subtilis* spores on lung-resident macrophages remain poorly understood. In this study, we aimed to delineate how alterations in spore coat architecture affect the immunostimulatory capacity of *B. subtilis*. By employing a panel of mutants deficient in specific coat proteins, we investigated their respective roles in modulating immune responses in pulmonary macrophage cell line MΦ3D4/2.

*B. subtilis* spores have been shown to activate human monocyte-derived macrophages, inducing the production of a range of pro-inflammatory cytokines and chemokines, including IL-1β, IL-6, and TNF-α, thereby modulating host immune responses [46]. Consistent with these findings, we observed that wild-type *B. subtilis* PY79 spores significantly elevated the expression of IL-1β, IL-8, and TNF-α in lung-resident macrophages (MΦ3D4/2). Notably, this immune activation was mediated through the MyD88-dependent signal pathway [44], a core adaptor utilized by nearly all Toll-like receptors (TLRs) except TLR3, leading to the production of inflammatory cytokines [47]. In contrast, spores derived from mutants lacking specific coat proteins exhibited distinct immunomodulatory profiles. All mutants significantly upregulated TLR4 expression, an observation that diverges from prior reports, which did not implicate TLR4 as a major receptor in *B. subtilis*-induced immunity [48]. This suggests that spore coat protein alterations may redirect pattern recognition pathways. Specifically, the *cotX*^−^ and *cotZ*^−^ mutants induced significant increases in TLR2 expression, which serves as a key interface between innate and adaptive immunity [49,50,51]. This points to CotX and CotZ as potential modulators of TLR2-mediated signal pathway. Moreover, both *cotX*^−^ and *cgeA*^−^ mutants enhanced TLR9 expression, implicating these proteins in the repression of TLR9-dependent pathways. TLR9 plays a pivotal role in sensing internal and external danger signals and maintaining immunological homeostasis [52]. These findings suggest that the spore crust exerts a suppressive effect on immune recognition, and its disruption, particularly in *cotX*^−^, *cgeA*^−^, and *cotZ*^−^ mutants, potentiates the activation of TLR2, TLR4, and TLR9 pathways.

We further observed that both *cotX*^−^ and *cotE*^−^ mutants strongly upregulated the transcription of MyD88 and NF-κB2, leading to enhanced IL-8 expression via the classical MyD88-dependent pathway. MyD88 and NF-κB2 have been well-established as crucial mediators in the immune modulation induced by *B. subtilis* [53,54]. In contrast, the *cgeA*^−^ mutant displayed a more generalized inflammatory response, significantly increasing NF-κB2 transcription and the expression of multiple pro-inflammatory cytokines, including IL-1β, IL-6, IL-8, and TNF-α. Although the *cotZ*^−^ mutant did not significantly alter NF-κB2 transcription, it still induced the production of IL-1β, IL-8, and TNF-α, suggesting that the *cotZ^−^* mutation might engage a MyD88-independent signaling pathway. Furthermore, disruption of the spore crust, most notably in the absence of CotX and SafA, resulted in an elevation of IRAK1 gene transcription. IRAK proteins are key regulators of IL-1R and TLR signaling pathways [55], both of which are crucial for modulating innate immunity and inflammation [56,57].

These findings provide compelling evidence for the inhibitory role of the spore crust in immune responses, particularly through proteins such as CotX, CotE, CotZ, and CgeA. The broad upregulation of pro-inflammatory cytokines such as IL-1β, IL-8, and TNF-α in multiple mutants suggests a generalized inflammatory response [58,59]. Notably, IL-8, a cytokine implicated in *B. subtilis*-induced neutrophil recruitment, was further enhanced in the mutants, potentially indicating that alterations in the spore coat enhance the immunostimulatory properties of vegetative cells or their derivatives [58]. Conversely, the lack of significant changes in IL-10 expression suggests that these mutations predominantly drive pro-inflammatory responses, with minimal effects on anti-inflammatory pathways. This finding contrasts with previous observations, highlighting a differential immune activation profile, which might be caused by the different strains of *B. subtilis* selected and the differences in the selected macrophage cell lines [60].

Overall, these findings highlight the indispensable roles of both inner and outer spore coat proteins in orchestrating host immune responses. Future investigations should dissect the specific immunomodulatory functions of individual coat components, providing mechanistic insights critical for the rational engineering of *Bacillus subtilis* spores as next-generation immunostimulatory agents. Moreover, extending these studies to diverse in vivo models—including murine, avian, bovine, and potentially human systems—will be essential to substantiate their probiotic efficacy and translational potential.

## 5. Conclusions

In this study, we delineate the immunostimulatory consequences of targeted deletions in key spore coat proteins (SpoIVA, SafA, CotE, CotX, CotZ and CgeA) in *Bacillus subtilis* spores. Immune modulation by these mutant spores was predominantly mediated via the TLR4 signal axis, with additional activation of TLR2 and TLR9 pathways observed in select mutants. Disruption of the crust layer, particularly in the absence of CotX, was associated with enhanced transcription of key immune signal mediators, including MyD88, NF-κB2, and IRAK1. Strikingly, mutant spores elicited robust upregulation of pro-inflammatory cytokines, such as IL-1β, IL-8, and TNF-α, underscoring the heightened immunogenicity resulting from alterations in spore coat integrity. These findings suggest that the crust layer may serve to attenuate host immune responses, acting as a modulatory interface. Together, our results provide new mechanistic insights into the immunological roles of spore coat components and establish a foundation for the rational design of *B. subtilis*-based probiotics with tailored immunomodulatory properties for agricultural and therapeutic use.

## Figures and Tables

**Table 1:** Primers employed in this study.

Primer	Sequence	Restriction Enzymes
*Clw-up*	ACCGAATTCGGGCATTGTTTCATC	*Eco*RI
*Clw-down*	TTGGATCCGAAATATGATTACCGCATG	*Bam*HI
*GerD-up*	GCGGATCCAAGCTGTTACAGATAATAA	*Bam*HI
*GerD-down*	GCGGAGCTCCAAAAAATAAAAAACGCAC	*Sac*I
*Specb-up*	TTGGATCCGAATGGCGATTTTCG	*Bam*HI
*Specb-dow*	GCCGGATCCTTCCACCATTTTTTC	*Bam*HI
*spoIVA*-fro-up	TTGAATTCTACGATGCTTTCTGCAATTG	*Eco*RI
ovlapIVA-1	ATTTTCTAAAGATGTGGATCCCGGTAGACCTC	*Bam*HI
ovlapIVA-2	AAAGAGGTCTACCGGGATCCACATCTTTAGAAAATTTC	*Bam*HI
*spoIVA*-bac-down	TTGAGCTCCATGTGTATGCTCATATCTGG	*Sac*I
*cotX*-fro-up	TTGCGAATTCGAACAGCAGATCGAAG	*Eco*RI
ovlapcotX-1	CTTTTAGGTCCTAGGATCCTGAGCGAGCCTC	*Bam*HI
ovlapcotX-2	ATAAGAGGCTCGCTCAGGATCCTAGGACCTAAAAG	*Bam*HI
*cotX*-bac-down	TTGAGCTCGCAGATCTTCAATATTTTCTAC	*Sac*I
*cgeA*-fro-up	TTGAATTCGGATGCACGAAACTGTTATG	*Eco*RI
ovlapcgeA-1	TCACAATGCTGATTGTGGATCCTACACACACCTC	*Bam*HI
ovlapcgeA-2	GGAGGTGTGTGTAGGATCCACAATCAGCATTG	*Bam*HI
*cgeA*-bac-down	GCCGAGCTCTTGATAGTAGAGAGCTGC	*Sac*I
*cotZ*-fro-up	TTGAATTCCTGCCCGCTAAGCAGGATC	*Eco*RI
ovlapcotZ-1	CAGGAGGGATAATGGATCCTCATAAGCTGGAAA	*Bam*HI
ovlapcotZ-2	TTTCCAGCTTATGAGGATCCATTATCCCTCCTGC	*Bam*HI
*cotZ*-bac-down	TTGAGCTCCGGCAACTCTGACATCAATTG	*Sac*I
*SafA*-fro-up	TTGAATTCCGCGCTTTGCATCCTGTG	*Eco*RI
ovlapSafA-1	GTTGAAAATCCATATGGATCCCGTTCGGAACGATGTAA	*Bam*HI
ovlapSafA-2	TACATCGTTCCGAACGGGATCCATATGGATTTTCAA	*Bam*HI
*Saf*A-bac-down	TTGAGCTCCGATGAAAGATGAATTAGTAGC	*Sac*I
*cotE*-fro-up	TTGAATTCAGAGACTCGCAAATGGAAG	*Eco*RI
*cotE*-fro-down	TTGGATCCTTCCAATTTTTTCAGCGTC	*Bam*HI
*cotE*-bac-up	TTGGATCCTAAAAAAGGGACTAGGGGAG	*Bam*HI
*cotE*-bac-down	TTGAGCTCCTCCAGATTACGCTTTGAG	*Sac*I

## Data Availability

The data presented in this study are available on request from the corresponding author.

## Supplementary Materials

The following supporting information can be downloaded at: https://www.mdpi.com/article/10.3390/cimb47050378/s1.
