# Supplementary material for: Disruption of Spore Coat Integrity in Bacillus subtilis Enhances Macrophage Immune Activation
Source: Curr Issues Mol Biol. 2025 May 20;47(5):378. doi: 10.3390/cimb47050378 (PMC12110675; doi:10.3390/cimb47050378)
Supplement: Supplementary file 1 [file cimb-47-00378-s001.zip › Supplementary Tables.pdf]

Table S1. Lengths of upstream and downstream homology arms used for mutant construction.

Homology arm	Length(bp)
spoIVA-fro/bac	126/931
cotE-fro/bac	590/934
cotX-fro/bac	771/858
cotZ-fro/bac	915/1013
SafA-fro/bac	939/648
cgeA-fro/bac	807/546

Table S2. Lengths of homologous recombination target fragments for each spore coat protein gene deletion.

Target fragment	Length(bp)
spoIVA	3657
cotE	2064
cotX	2149
cotZ	2459
SafA	2751
cgeA	1757

Table S3. Primer sequences employed in qRT-PCR assays.

Primer	Sequence
GAPDH-F	ACATCATCCCTGCTTCTACTGG
GAPDH-R	CTCGGACGCCTGCTTCAC
TLR2-F	GCCAATAGCATTCATACCG
TLR2-R	GATTCGCAGATTTGGGAGA
TLR4-F	GGCATCATCTTCATCGTCC
TLR4-R	CTCCCACTCCAGGTAGGTATT
TLR9-F	CGACTGCATCACCAAGACCA
TLR9-R	TGCATGTCCAGCTCCTTCAG
NF-κB2-F	TCCGATTCCGATATGGCTGC
NF-κB2-R	TGTGGGCATGAGCACGAG
IRAK1-F	CAACAGCATCTCTGCACCCT
IRAK1-R	AATGAGTCTGGGAGCCTGGA
MyD88-F	GCGGAGGAGATGAACTTCGAG
MyD88-R	ATACTTTCGGCAGTCCTCTTCA
