# Supplementary figures and images for: Disruption of Spore Coat Integrity in Bacillus subtilis Enhances Macrophage Immune Activation
Source: Curr Issues Mol Biol. 2025 May 20;47(5):378. doi: 10.3390/cimb47050378 (PMC12110675; doi:10.3390/cimb47050378)

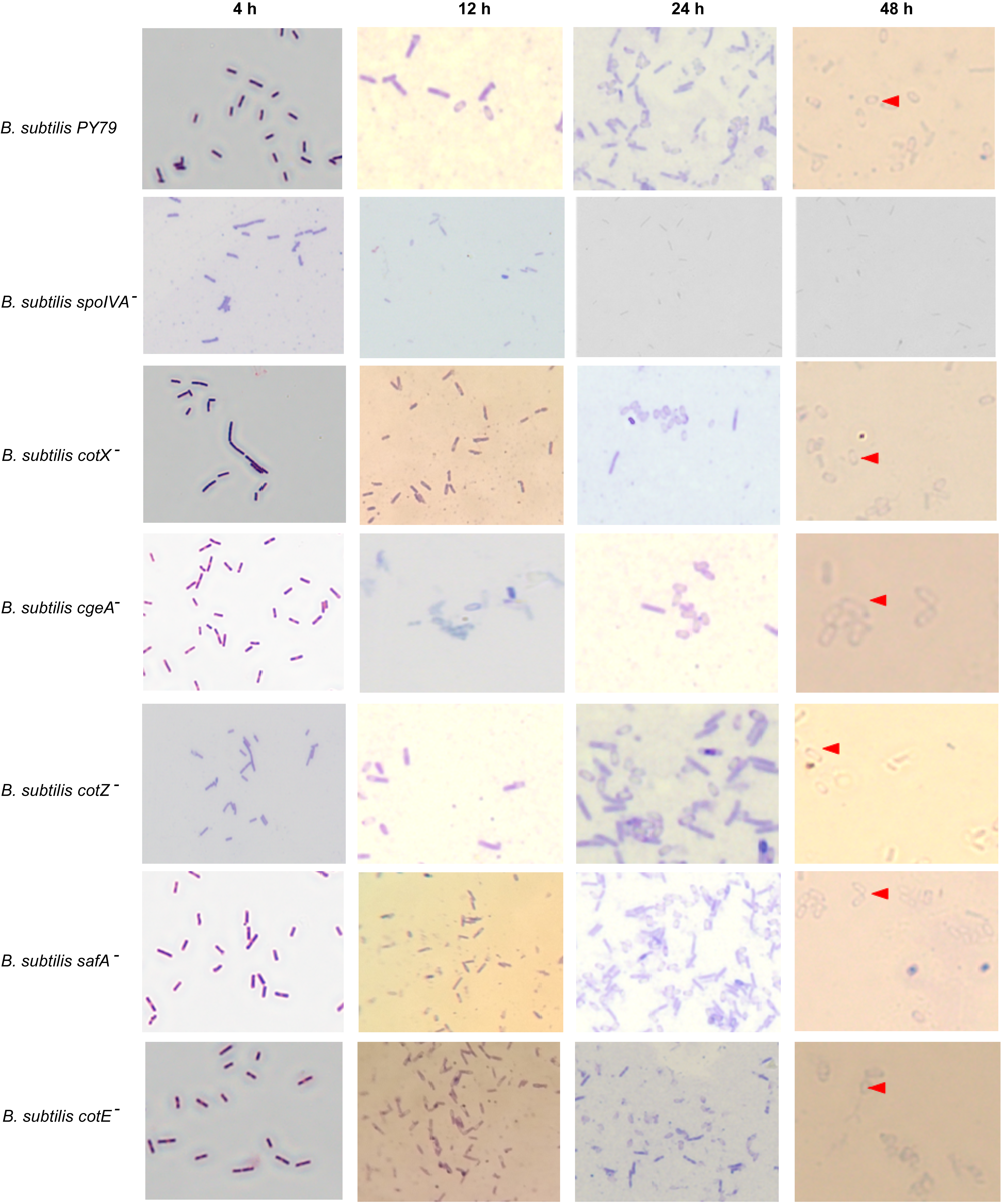

Supplement: Supplementary file 1 [file cimb-47-00378-s001.zip › Figure S1.tif]
